# *Fasciola hepatica*: effect of the natural light level on cercarial emergence from temperature-challenged *Galba truncatula*

**DOI:** 10.1051/parasite/2014009

**Published:** 2014-02-28

**Authors:** Philippe Vignoles, Amal Titi, Daniel Rondelaud, Abdeslam Mekroud, Gilles Dreyfuss

**Affiliations:** 1 INSERM 1094, Faculties of Medicine and Pharmacy 87025 Limoges France; 2 PADESCA Laboratory, Veterinary Science Institute, University Constantine 1 25100 El Khroub Algeria

**Keywords:** Cercaria, Cercarial emergence, Light, *Fasciola hepatica*, *Galba truncatula*, Temperature

## Abstract

As abrupt changes in water temperature (thermal shock) triggered a significantly greater cercarial emergence of *Fasciola hepatica* from experimentally infected *Galba truncatula*, laboratory investigations were carried out to study the influence of light on cercarial emergence in snails subjected to a thermal shock every week (a mean of 12 °C for 3 h) during the patent period. Thermal shock for these temperature-challenged (TC) snails was carried out outdoors under artificial or natural light, or indoors under constant artificial light. Compared with the infected control snails always reared indoors at 20 °C, the number of cercariae in TC snails subjected to a thermal shock and natural light outdoors was significantly greater. The repetition of this experiment by subjecting TC snails to the same thermal shock indoors under an artificial light level ranging from 600 to 3000 lux did not show any significant difference among the numbers of cercariae in the different subgroups. A detailed analysis of the results noted in the TC snails subjected to natural light during the thermal shock demonstrated that the number of cercariae-releasing snails was significantly higher between 601 and 1200 lux and for the highest nebulosity values (7–8 octas, which corresponds to a sufficiently or completely overcast sky). Contrary to the intensity of artificial light, which did not influence cercarial emergence, the natural light level had a significant effect on this process when *F. hepatica*-infected snails were subjected to a regular thermal shock during the patent period.

## Abstract (fr)

Comme de brusques changements dans la température de l’eau (choc thermique) provoquent des émissions cercariennes de *Fasciola hepatica* significativement plus importantes chez des *Galba truncatula* infestés expérimentalement, des investigations ont été réalisées au laboratoire pour étudier l’influence de la lumière sur l’émergence des cercaires chez des mollusques soumis chaque semaine à un choc thermique (12 °C en moyenne pendant 3 heures) au cours de la période patente. Pour réaliser ce choc thermique, des limnées infestées ont été placées à l’extérieur (sous une lumière naturelle au artificielle) ou à l’intérieur (sous un niveau constant de lumière artificielle). Par rapport à des témoins infestés toujours élevés au laboratoire à 20 °C, le nombre de cercaires chez des limnées soumises à un choc thermique extérieur sous une lumière naturelle est significativement plus élevé. La répétition de cette expérience avec des limnées soumises à un choc thermique à l’intérieur, sous une lumière artificielle allant de 600 à 3000 lux ne montre pas de différence significative entre les nombres de cercaires pour les différents sous-groupes. Une analyse détaillée des résultats obtenus chez les limnées soumises à un choc thermique extérieur sous lumière naturelle montre que le nombre de mollusques émettant des cercaires est significativement plus élevé entre 601 et 1200 lux ainsi que pour les valeurs les plus élevées de la nébulosité (7–8 octas, ce qui correspond à un ciel suffisamment ou complètement bouché). À l’inverse de l’intensité de la lumière artificielle qui n’a pas d’influence sur l’émergence des cercaires, le niveau de la lumière naturelle a un effet significatif sur ce processus lorsque les mollusques infestés par *F. hepatica* sont soumis à un choc thermique régulier au cours de la période patente.

## Introduction

The emergence of infective trematode stages (cercariae) from their intermediate hosts is often initiated in response to environmental cues. Temperature is the most important factor governing cercarial emergence. According to digenean species, the emergence of cercariae from their snail hosts may be triggered by increasing or decreasing the temperature so that the effect of this factor is often trematode-specific [30]. Other abiotic factors such as light or water pH may also have an impact on initiating the exit of cercariae. Light, for example, has been demonstrated to be an important factor in some snail-parasite models and particularly for schistosomes such as *Schistosoma haematobium* [5, 17, 19], *S. intercalatum* [15, 16] and *S. mansoni* [31–33], so that different schistosome species showed strikingly different circadian patterns of emergence corresponding with light-dark cycles [3]. According to Koprivnikar and Poulin [12], it is important to identify external factors controlling the emergence of cercariae in order to understand trematode transmission and the maintenance of complex trematode life cycles.

In the model *Fasciola hepatica*-*Galba truncatula*, the stimulating effect of decreasing temperature on cercarial emergence had already been reported by Kendall and McCullough [11]. This factor was often used by subsequent authors to induce this process by subjecting cercariae-containing snails to abrupt changes in temperature, followed by an increase [10, 13, 23]. Contrary to temperature, the effect of daylight is more conflicting. Kendall and McCullough [11] did not find any significant effect of illumination on the emergence of *F. hepatica* cercariae and noted that emergence occurred equally under conditions of light or darkness. According to these authors, the emergence of cercariae during the day or night was directly associated with the management of infected snails (water change) under observation. Despite this result, subsequent authors such as Hodasi [10] have used strong illumination of infected *G. truncatula* to induce cercarial emergence and several have reported that cercariae in the field emerged from snails during bright sunny days [6, 8]. According to Malone and Yilma [13], such differences might be explained by environmental differences between regions and possible intrinsic biological differences in the *F. hepatica*-snail host system.

In view of this discrepancy between the above results, it would be interesting to determine if natural fluctuations in daylight may have a stimulating effect on *F. hepatica* cercarial emergence. As an abrupt change in water temperature (thermal shock) triggered a significantly greater cercarial production [23], the response to this question may be solved by studying the effect of natural or artificial light on snails already subjected to these weekly temperature challenges. Experimental infections of *G. truncatula* with *F. hepatica* were thus carried out and cercarial emergence was followed outdoors during these weekly abrupt changes in water temperature in relation to the natural light level. The same operation was also performed indoors by exposing temperature-challenged snails to different levels of artificial light.

## Materials and methods

### Snails and parasite

The two populations of *G. truncatula* were living in road ditches in the communes of Thenay (46°37′23″ N, 1°26′12″ E) and Chitray (46°40′31″ N, 1°22′17″ E), in the department of Indre (central France). They were selected because of the particular location of their habitats. The road ditch in Thenay was situated inside a forest and only received sunlight during several hours in the afternoon. In contrast, the snail habitat in Chitray was bordering a cattle-grazed meadow without tree covering and so received sunlight during most of the day. These snail habitats were selected for this study because of their high susceptibility to experimental infections with *F. hepatica* [24] and their different ecological exigencies in order to determine if the influence of light during thermal shock on cercarial emergence was a general phenomenon in central France, whatever the snail population studied. As these two sites were located on a soil with predominant clay, the maximum height of adult snails ranged from 11 to 12 mm. Two hundred snails (Table 1), measuring 4 ± 0.1 mm in height and belonging to the overwintering generation, were collected from the ditch located in Thenay, while 500 others with the same shell height and belonging to the spring generation came from the habitat located in Chitray. Thirty adult snails were also picked up from each site and were dissected under a stereomicroscope to verify the absence of trematode larval forms within their body. After their collection, snails were kept in the laboratory at 20 °C for 48 h for temperature acclimatisation before being exposed to miracidia.

**Table 1.** Main characteristics of snail subgroups used in the two experiments. To induce thermal shock, Petri dishes containing snails were exposed to a mean of 12 °C for 3 h every week. TC: temperature-challenged snails.

Experiment, snail origin and number of snails	Characteristics of subgroups at day 30 post-exposure	Number of snails	
		At day 30	Releasing cercariae
Experiment A			
Thenay (200)	Controls kept indoors at 20 °C under a 3000-lux artificial light	48	37
	TC snails subjected to a thermal shock outdoors under:		
	a constant artificial light of 3000 lux*	49	33
	natural light*	49	39
Experiment B			
Chitray (200)	Controls kept indoors at 20 °C under a 3000-lux artificial light	43	30
	TC snails subjected to a thermal shock outdoors under:		
	a constant artificial light of 3000 lux*	43	37
	natural light*	44	34
			
Chitray (300)	TC snails subjected to a thermal shock indoors under a constant artificial light level of:		
	600 lux*	39	29
	1200 lux*	39	33
	1800 lux*	39	38
	2400 lux*	39	32
	3000 lux*	39	35

* The rest of the week, the TC snails of the nine subgroups were placed indoors at a constant temperature of 20 °C as for controls.

Eggs of *F. hepatica* came from the gall bladders of heavily infected cattle at the slaughterhouse of Limoges, in the department of Haute Vienne. These eggs were washed several times with spring water and were incubated at 20 °C for 20 days in the dark to obtain miracidia [14].

### Snail infection and breeding

Two experiments (Table 1) were carried out from January to July 2012. In experiment A, the cercarial emergence of *F. hepatica* was studied in snails subjected to a thermal shock every week (temperature-challenged snails) outdoors in relation to the natural light level, while infected snails raised under constant conditions were used as controls. Experiment B was done to study cercarial emergence from temperature-challenged (TC) snails maintained indoors under constant levels of artificial light.

In experiment A, two hundred snails, originating from the population of Thenay, were individually exposed to *F. hepatica* (two miracidia per snail for 4 h at 20 °C in 3.5 mL of spring water). A similar protocol was used for the other 200 snails from the population of Chitray (Table 1). Snails were then individually raised in 35-mm Petri dishes (volume of spring water, 3.5 mL) according to the method of Rondelaud et al. [21]. Snails were fed with a piece of dried lettuce leaf and another of dead grass (*Molinia caerulea*), while oxygenation of the water layer was ensured by a piece of live spring moss (*Fontinalis* sp.). The dissolved calcium in the spring water was 35 mg/L. The Petri dishes were placed in an air-conditioned room at a constant temperature of 20 °C (±1 °C) and a diurnal photophase of 10 h involving a light of 3000 lux. At day 30 post-exposure (p.e.), the surviving snails from Thenay were divided into three subgroups. The first subgroup was constituted snails which were always maintained indoors at a constant temperature of 20 °C under a 3000-lux artificial light and were not subjected to a regular thermal shock (see below). Snails of this first subgroup were considered as controls in the case of Thenay. The other two subgroups were subjected to a thermal shock every week (temperature-challenged snails) by placing Petri dishes containing snails outdoors under a constant artificial light level of 3000 lux (3000 lux subgroup) or natural light (Table 1). A similar protocol was also used for the surviving *G. truncatula* from Chitray (Table 1).

In experiment B, three hundred *G. truncatula* originating from the population of Chitray were also subjected to bimiracidial infections. Snails living in Thenay were not involved in this experiment because their habitat (a road ditch) was cleaned in spring just after the collection of the first 200 snails used in experiment A and the number of surviving snails was at that time too low to do experimental infections. Snail infection and breeding during the first 30 days of the experiment were similar to those used in experiment A. At day 30 p.e., the surviving snails were divided into five subgroups, as indicated in Table 1. These five subgroups were required in order to subject these snails to a thermal shock every week, but indoors under a constant artificial light level of 600, 1200, 1800, 2400 or 3000 lux, respectively. Each subgroup was subjected to the same constant level of artificial light during the days of thermal shock throughout the experiment. For this, they were placed indoors in a ventilated box (150 × 120 × 150 cm) but subjected to the outdoor temperature as in experiment A. In each box, a 14 W Grolux fluorescent tube and a dimmer switch allowed a constant level of artificial light.

Apart from the time passed to subject snails to the thermal shock every week, the temperature-challenged (TC) snails in the two subgroups of Thenay, the two others of Chitray in experiment A, and the five subgroups in experiment B were maintained at a constant temperature of 20 °C during the rest of the week. The diurnal photophase was 10 h and the light level was 3000 lux, as for the two control subgroups in experiment A. In all the snail subgroups, spring water and food, if necessary, were changed daily between 4 and 7 p.m.

### Thermal shock and light level

When the first cercarial emergence occurred, the Petri dishes containing snails of nine TC subgroups (four in experiment A and five in experiment B) were placed every week at a mean temperature of 12 °C (minimum-maximum, 10–14 °C) for 3 h (from 8 to 11 a.m.). The mean temperature of 12 °C was chosen because most cercariae in the case of *F. hepatica* did not emerge from snails below 10 °C [1]. The reason for selecting a weekly interval for temperature changes was the 6–8-day periodicity that Vignoles et al. [34] reported in cercarial emergence for some snails infected with *F. hepatica*. To induce thermal shock, the morning was chosen because the water temperature in the Petri dishes fell from 20 °C to 10–11 °C in 20–25 min (when these recipients were placed at this temperature at 8 a.m.) and progressively increased to 14 °C with increasing air temperature (generally reached at 11 a.m.). The above protocol was followed for 11 weeks in the case of *F. hepatica* according to the length of the patent period.

In experiment A, the Petri dishes subjected to thermal shock were placed outdoors and protected from direct sunshine by putting them under a sloping roof (TC snails, 3000 lux) or in the shade if necessary (TC snails, natural light) The natural light level was measured each hour during the time of thermal shock (3 h). Sky nebulosity (this parameter expresses the extent of the cloud covering, going from 0 octa for a clear sky to 8 octas for a completely overcast sky) was also taken into account from data furnished by the meteorological station of Limoges-Bellegarde. The light level was measured using a MT0 001 Light Lux Meter (Velleman components) just above the Petri dishes.

After the replacement of the Petri dishes containing TC snails at 20 °C, cercariae exited from the snails in the following 2–3 h and were counted each day during the water and food change (between 4 and 7 p.m.). If cercariae were present in a Petri dish, the snail and its food were placed in a second dish, and the larvae of the first dish were counted two days later before their removal.

### Parameters studied

The first two parameters noted in each snail subgroup were (i) the number of cercariae during the days with a thermal shock (TC snails) or the corresponding days for controls always maintained at 20 °C (experiment A) and (ii) their frequency using the ratio: number of cercariae during the days with a thermal shock (or the corresponding days for controls)/total number of cercariae emerged during the patent period. The third parameter was the time interval between the end of the thermal shock and the beginning of cercarial emergence.

As the best results on cercarial production were noted in the two snail subgroups subjected to natural light during the thermal shock (experiment A), these data were also analysed in relation to the mean level of natural light and sky nebulosity. The mean level of natural light (calculated from light measurements performed from 8 to 11 a.m. during each day with a thermal shock) was expressed in 600-lux classes. Sky nebulosity was expressed in 2-octa classes. For each light class and each nebulosity class, the two parameters were the number of cercariae-releasing (CR) snails and the number of emerged cercariae.

Individual values recorded for the number of CR snails, that of cercariae, and the time interval between the end of the thermal shock and the first cercarial emergence were averaged and standard deviations were calculated, taking into account the snail subgroup (in both experiments), natural light class and sky nebulosity class (in the two TC subgroups subjected to natural light level during thermal shock in experiment A).

### Data analysis

As the aim of the present study was to determine the influence of the light level on cercarial emergence, the differences noted in experiment A between the numbers of cercariae or time intervals (TC snails only) were analysed for each snail population considered separately. In experiment B, a similar protocol was used for the values noted for the numbers of cercariae and time intervals.

In the two snail subgroups subjected to natural light during thermal shock (experiment A), the influence of the light level and sky nebulosity on cercarial emergence was analysed by comparing differences between the numbers of CR snails or the quantities of emerged cercariae for each class of light level and each class of sky nebulosity. The influence of each climatic factor on these parameters was analysed separately because the classes of light level did not correspond to those defined for nebulosity (two or three close light classes were noted for the same class of nebulosity during the experiment).

In experiments A and B, the frequencies of cercariae emerged during the days with a thermal shock were subjected to a *χ* ^2^ test. The normality of values concerning CR snails and cercariae was analysed using the Shapiro-Wilk normality test [29]. As their distribution was non-normal, the Kruskal-Wallis test was used to establish levels of significance. Differences between time intervals were subjected to a Student *t* test. All the statistical analyses were done using Statview 5.0 software (SAS Institute Inc., Cary, NC, USA).

## Results

### Type of light used during thermal shock and cercarial emergence

Table 2 gives the results of both experiments. In each snail population considered separately (experiment A), the frequency of cercariae noted during the days after the thermal shock (Table 2) was significantly higher (Thenay: *χ* ^2^ = 1650.08, *p* < 0.001; Chitray: *χ* ^2^ = 1430.61, *p* < 0.001) in the TC snails subjected to natural light than in controls and TC snails subjected to a 3000-lux artificial light. The same finding was also noted for the number of cercariae per CR snail, with a significant difference between controls and TC snails (Thenay: *H* = 69.63, *p* < 0.001; Chitray: *H* = 68.89, *p* < 0.001). Time intervals between the end of the thermal shock and the beginning of cercarial emergence were significantly shorter (Thenay: *t* = 3.45, *p* < 0.01; Chitray: *t* = 6.21, *p* < 0.001) in TC snails subjected to natural light. In experiment B, the frequencies of cercariae emerged during the days after the thermal shock ranged from 54.9% to 57.7% and no significant difference among the five subgroups was recorded. The same finding was also noted for the numbers of cercariae and the time intervals between the end of the thermal shock and the beginning of cercarial emergence, whatever the mode of comparison.

**Table 2.** Numbers of *Fasciola hepatica* cercariae counted during days of thermal shock for temperature-challenged (TC) snails and the corresponding days for controls, with indication of time intervals between the end of thermal shock and the beginning of cercarial emergence. The number of cercariae-releasing (CR) snails in each subgroup is given in Table 1.

Snail subgroups	Cercariae of *F. hepatica*			
	Total number	Frequency (%)	Mean number (*SD*) per CR snail	Mean time interval (*SD*)
Experiment A (thermal shock of TC snails done outdoors)				
Thenay, controls	3306	57.78	89.3 (41.2)	–
Thenay, TC snails:				
3000-lux artificial light	6746	78.58	204.4 (71.9)	3 h 02 min (57 min)
natural light	11,673	84.45	299.3 (81.2)	1 h 45 min (35 min)
Chitray, controls	2289	43.92	76.3 (37.9)	–
Chitray, TC snails:				
3000-lux artificial light	6612	57.75	178.7 (58.3)	3 h 37 min (41 min)
natural light	9809	72.39	288.5 (76.0)	1 h 21 min (24 min)
Experiment B (thermal shock of TC snails done indoors under artificial light)				
Chitray, 600 lux	4886	54.93	168.4 (47.2)	3 h 13 min (46 min)
Chitray, 1200 lux	6507	56.11	197.1 (72.8)	2 h 56 min (16 min)
Chitray, 1800 lux	6137	55.27	161.5 (54.7)	3 h 2 min (29 min)
Chitray, 2400 lux	5040	57.76	157.5 (45.1)	3 h 21 (35 min)
Chitray, 3000 lux	5995	55.08	171.2 (64.3)	3 h 11 min (32 min)

### Natural light level, sky nebulosity and cercarial emergence

The data noted in the TC subgroups subjected to natural light during thermal shock (experiment A) are given in Table 3 in relation to the light level and in Table 4 in relation to sky nebulosity. In both populations (Table 3), the highest numbers of CR snails were noted in the 601–1200 lux class and were significantly greater (Thenay: *H* = 51.20, *p* < 0.001; Chitray: *H* = 32.93, *p* < 0.001) than those recorded in upper lux classes. In contrast, the differences among the numbers of cercariae were not significant, whatever the mode of comparison. When the sky was overcast (7–8 octas, Table 4), the numbers of CR snails were significantly more numerous (Thenay: *H* = 52.31, *p* < 0.001; Chitray: *H* = 53.76, *p* < 0.001) than in the other classes. Non-significant differences among these numbers of cercariae were noted. The natural light level and sky nebulosity, associated with thermal shock, seem to have a significant influence on cercarial emergence by stimulating this process.

**Table 3.** Number of cercariae-releasing (CR) snails and number of *Fasciola hepatica* cercariae in the two temperature-challenged subgroups subjected to natural light during thermal shock (experiment A) in relation to light level expressed in 600-lux classes each.

Natural light level (lux)	Thenay			Chitray		
	Total number of cercariae (*n* = 11,673)	Number of CR snails (*n* = 39)*	Number of cercariae per CR snail*	Total number of cercariae (*n* = 9809)	Number of CR snails (*n* = 34)*	Number of cercariae per CR snail*
1–600	2764	8.9 (5.2)	310.5 (71.9)	1902	7.1 (4.7)	267.8 (100.2)
601–1200	4468	13.8 (8.1)	323.7 (95.6)	4510	12.7 (9.2)	355.1 (135.6)
1201–1800	1712	6.4 (4.1)	267.5 (43.3)	1432	5.7 (3.0)	251.2 (85.3)
1801–2400	1121	4.1 (2.7)	273.4 (51.0)	926	4.0 (2.6)	231.5 (55.1)
2401–3000	901	3.3 (1.6)	273.0 (62.3)	600	2.4 (1.6)	250.0 (63.0)
>3000	707	2.5 (1.4)	282.8 (47.5)	439	2.1 (1.3)	209.0 (41.3)

* Mean value (*SD*).

**Table 4.** Number of cercariae-releasing (CR) snails and number of *Fasciola hepatica* cercariae in the two temperature-challenged subgroups subjected to natural light during thermal shock (experiment A) in relation to sky nebulosity expressed in 2 octas each.

Sky nebulosity (octas)	Thenay			Chitray		
	Total number of cercariae (*n* = 11,673)	Number of CR snails (*n* = 39)*	Number of cercariae per CR snail*	Total number of cercariae (*n* = 9809)	Number of CR snails (*n* = 34)*	Number of cercariae per CR snail*
0	356	1.8 (0.7)	197.7 (23.6)	251	1.4 (0.5)	179.2 (41.5)
1–2	1352	5.1 (2.3)	265.0 (41.7)	692	2.6 (1.1)	266.1 (78.3)
3–4	1878	6.4 (4.2)	293.4 (63.7)	1320	5.1 (2.9)	258.8 (94.6)
5–6	2388	9.3 (5.1)	256.7 (94.5)	2124	7.8 (4.5)	272.3 (77.4)
7–8	5699	16.4 (9.2)	347.5 (123.5)	5422	17.1 (10.4)	317.0 (114.7)

* Mean value (*SD*).

High numbers of cercariae, ranging from 157 to 341 per TC snail and day with a thermal shock, were frequently noted in the lowest classes of light level (from 1 to 1200 lux) and the upper classes of sky nebulosity (data not shown).

## Discussion

The results noted in experiments A and B demonstrate that natural light had a significant influence on cercarial emergence of *F. hepatica* when the infected snails were subjected to a regular thermal shock during the patent period. Even if the abrupt change in temperature during thermal shock constitutes the main factor to stimulate cercarial emergence of *F. hepatica* [23], natural light also plays a role in cercarial emergence. As these findings were noted in two snail populations, each having its own ecological exigencies, and two snail generations, the influence of this environmental factor on cercarial emergence of *F. hepatica* seems to be general, at least for the *G. truncatula* living in the lowlands of central France. In the case of TC snails subjected to natural light during thermal shock, the highest numbers of cercariae-releasing snails were noted in low light conditions (Table 3) and under a sufficiently or completely overcast sky (7–8 octas, Table 4). These results disagree with the reports by Ginetsinkaya [6] and Graczyk and Fried [8]. According to these authors, *F. hepatica* cercariae in the field emerged from snails during bright sunny days. To comment on this discrepancy with the above authors, two perhaps complementary explanations may be proposed. The first is to relate the finding reported in the present paper to environmental conditions (temperate climate) which exist in the sites of both populations. The second explanation is to relate this finding to the process of cercarial emergence which normally occurred in the field for *F. hepatica*-infected snails. As most cercariae of *F. hepatica* [2], like those of another fasciolid, *Fasciola gigantica* [4, 9] exited from their intermediate host during the night, the brutal decrease in temperature during the thermal shock, associated with dim natural light, constitute conditions which would be similar to those occurring at twilight in spring or autumn. These conditions would thus be sufficiently efficient to stimulate free cercariae within the body of snails and produce cercarial emergence during the day after the thermal shock (instead of the night according to Audousset et al. [2]). These conditions might also be advantageous for dispersal and widespread encystment of *F. hepatica* cercariae on vegetation.

In the two snail populations of experiment A, the numbers of cercariae noted in the TC subgroups subjected to natural light during thermal shock were significantly higher than those of controls and other TC snails (Table 2). This finding may be explained by the high numbers of cercariae released by the lymnaeid (from 157 to 341 per TC snail and per day with a thermal shock). In contrast, such concentrations of cercariae emerging during a day raise a problem because they were generally scarce under laboratory conditions and were observed before the death of infected snails when cercariae exited in mass from their intermediate host [6, 8, 22]. In the present study, these concentrations of cercariae are all the more surprising since several snails of these two TC subgroups had released such quantities of cercariae during two or three days of thermal shock (the shell height of these snails generally ranged from 7 to 8 mm during the patent period) and apparently did not suffer from such cercarial emergence in their physiological state. These data demonstrate that natural light, associated with the use of thermal shock, allow the significant enhancement of cercarial emergence of *F. hepatica* from infected snails, and this process might be routinely used for commercial production of these digenean infective stages.

The time interval which exists between the end of thermal shock and the beginning of cercarial emergence was noted in all experiments that our team carried out by using this method to stimulate cercarial emergence of digeneans from their snail host [23, 25, 27, 28]. It was also reported by Kendall and McCullough [11] between the daily water change for infected snails and cercarial emergence (a time interval of 2 or 3 h). In our opinion, this time interval would be used by free cercariae to dig galleries through the snail’s perianal region before their exit into water [22]. Under these conditions, it was difficult to understand why there was a significantly shorter time interval in the two TC subgroups exposed outdoors to natural light during thermal shock, whereas these times in the other two TC subgroups of experiment A and in those of experiment B (Table 2) did not significantly differ from each other. The stimulating action of refreshed water on cercarial emergence [11, 13] cannot be accepted here because daily water changes were carried out in our experiments at the end of afternoon, just after the emergence of the last cercariae. As the conditions of snail maintenance in Petri dishes, except for the quality and quantity of light, were the same for all TC snails, the factor responsible for these shorter intervals in the two subgroups exposed to natural light during thermal shock can only be due to a particular (or several close) wavelength(s) existing in the spectrum of natural light. This (or these) wavelength(s) during thermal shock of TC snails would stimulate the behaviour of free cercariae and their emergence from the infected snails. Such effects of these wavelengths on cercarial behaviour have already been reported, for example, by Rea and Irwin [19] for *Cryptocotyle lingua* and by Rosen et al. [26] for *Proterometra macrostoma*. In the case of *F. hepatica*, these effects have only been analysed for miracidia [7, 20] and, to our knowledge, do not seem to have been studied for cercariae. Additional experiments are still necessary to specify the wavelength(s) of natural light which would stimulate cercarial behaviour in the body of infected *G. truncatula* and to determine if this (these) wavelength(s) is (are) identical to those reported by the above authors for *F. hepatica* miracidia.

In conclusion, the natural light level had a significant effect on cercarial emergence of *F. hepatica* when the infected snails were subjected to a regular thermal shock during the patent period. Low light levels increased cercarial production, while time intervals between the end of thermal shock and the beginning of cercarial emergence were shorter. These first findings need to be confirmed by determining if the effect of the natural light level on cercarial emergence exists in other French populations of infected *G. truncatula* and by specifying the wavelengths which act on this process.
